# Secretomes From Non‐Small Cell Lung Cancer Cells Induce Endothelial Plasticity Through a Partial Endothelial‐to‐Mesenchymal Transition

**DOI:** 10.1002/cam4.70707

**Published:** 2025-03-03

**Authors:** Clara Bourreau, Emilie Navarro, Marine Cotinat, Morgane Krejbich, François Guillonneau, Catherine Guette, Alice Boissard, Cécile Henry, Isabelle Corre, Lucas Treps, Nicolas Clere

**Affiliations:** ^1^ Univ Angers, Inserm, CNRS, MINT, SFR ICAT Angers France; ^2^ Nantes Université, Université d'Angers, CHU Nantes, Inserm, CNRS, CRCI2NA Nantes France; ^3^ Institut de Cancérologie de l'Ouest Angers France

**Keywords:** EndMT, endothelial cell plasticity, endothelial‐to‐mesenchymal transition, non‐small cell lung cancer, secretome

## Abstract

**Aim:**

The tumor microenvironment (TME) of non‐small cell lung cancer (NSCLC) is highly heterogeneous and is involved in tumorigenesis and resistance to therapy. Among the cells of the TME, endothelial cells are associated with the latter processes through endothelial‐to‐mesenchymal transition (EndMT). During EndMT, endothelial cells (ECs) progressively lose their endothelial phenotype in favor of a mesenchymal phenotype, which favors the production of cancer‐associated fibroblasts (CAFs). Our study aimed to investigate the consequences of exposure to different lung tumor secretomes on EC phenotype and plasticity.

**Materials and Methods:**

Conditioned media (CM) were prepared from the tumor cell lines A549, H1755, H23, H1437, and H1975. Proliferation and migration of ECs treated with these CMs were assessed by Cyquant and Incucyte technologies, respectively. The angiogenic capacity of ECs was assessed by following tubulogenesis on Matrigel. Phenotypic changes in treated ECs were detected by flow cytometry. Morphological analysis of actin fibers was performed by immunohistochemistry, while proteomic analysis by mass spectrometry was used to identify the protein content of secretomes.

**Results:**

A change of the endothelial phenotype was found when human umbilical vein endothelial cells (HUVECs) were treated with different CMs. This phenotypic change was associated with a morphological change, an increase in both stress fiber expression and spontaneous migration. Furthermore, an increase in mesenchymal markers (α‐SMA and CD44) confirmed the phenotypic changes. However, the secretomes did not modify the rate of double‐labeled cells (vWF^+^/α‐SMA^+^ or CD31^+^/CD44^+^). Proteomic analysis identified potential targets involved in the EndMT with therapeutic relevance.

**Conclusion:**

Taken together, these data suggest that CMs can induce partial EndMT.

## 1 Introduction

Non‐small cell lung cancer (NSCLC) is the most common lung cancer, which accounts for 85% of all lung cancers [1, 2] Despite therapeutic advances, NSCLC has a poor prognosis with a 5‐year survival rate of 15% owing to treatment resistance and tumor progression [3]. NSCLC is characterized by a heterogeneous tumor microenvironment (TME). During the last decade, the implication of the TME has been recognized as a main actor in tumor progression, aggressiveness, and as a source of drug resistance [4]. This TME includes endothelial cells (ECs), cancer‐associated fibroblasts (CAFs), a characteristic extracellular matrix (ECM), neuronal cells, as well as infiltrating immune cells [5].

Endothelial cell are one of the main actors in angiogenesis, which is defined as the formation of new vessels from pre‐existing networks [6, 7]. This pathophysiological process is essential for tumor growth, migration, invasion of tumor cells, and metastatic dissemination. Largely exposed to cues from the TME, ECs can undergo various phenotypic changes, among which is the endothelial‐to‐mesenchymal transition (EndMT) [8, 9, 10]. The EndMT is a cell differentiation process that potentiates the acquisition of mesenchymal‐like properties with a reduced cell proliferation, morphological alterations (spindle shape and appearance of stress fibers, decrease of cell–cell adhesion and polarity), enhanced migration, and increased mesenchymal markers including α‐SMA (α‐smooth muscle actin), CD44, N‐cadherin or fibronectin [9, 11, 12]. In addition, EndMT‐transiting cells exhibit a decrease of endothelial markers such as VEGFR2, von Willebrand Factor (vWF) and CD31, and an impaired tube formation capacity [13]. Ultimately, the EndMT is believed to increase the invasive and metastatic dissemination properties of tumor cells through the conversion of ECs into CAFs [14, 15]. Despite this well‐established process, a partial EndMT has been reported, with transiting ECs expressing a mixed phenotype [16]. Because of their great plasticity, the EndMT phenomenon can be reversible or can only partially take place, resulting in the temporary and reversible appearance of cells with an intermediate phenotype that concomitantly displays endothelial and mesenchymal characteristics [16, 17]. In addition, various studies are beginning to highlight the involvement of partial EndMT in angiogenesis and that a disruption in its program could thus contribute to the abnormal and pathological growth of blood vessels [18, 19].

In lung, while EndoMT has been mostly studied in fibrosis, its characterization in NSCLC is still in its infancy [20, 21]. The aim of this study was to demonstrate how the secretome of different NSCLC tumor lines can influence EC plasticity, particularly about the EndMT.

## 2 Methods

### 2.1 Cell Culture and Treatment

NSCLC cell lines were obtained from the ATCC (LGC Standards): NCI‐H1755 [H1755], A549—CCL‐185, NCI‐H23 [H23]—CRL‐5800, NCI‐H1437 [H1437]—CRL‐5872, NCI‐H1975 [H1975]—CRL‐5908. They were grown in RPMI supplemented with 10% fetal bovine serum decomplemented (SVFd), 2 nM l‐glutamin, and 1% streptomycin/penicillin (PS).

Human umbilical vein endothelial cells (HUVECs) were obtained from LONZA (C2519A) and cultured in endothelial cell growth medium‐2 (EGM2; PromoCell, C‐22011B) supplemented with fetal bovine serum (0.02 mL/mL) and different growth factors (PromoCell, C‐39216). Cells were cultured on a 0.1% gelatin‐coated T75 flask at 37°C and 5% CO_2_ in a humidified chamber. HUVECs were used between the first and sixth passages.

Normal human bronchial epithelial cell line (BEAS‐2B, ATCC CRL‐3588) was cultured in airway epithelial cell basal medium (PCS‐300‐030) and bronchial epithelial cell growth kit (PCS‐300‐040) as per the recommendations.

Given that all NSCLC lines have not the same doubling time, and to ensure reproducibility, a specific cell number was seeded for every type of tumor cell line, so that 70%–90% of confluence is reached at the end of the experiment. Tumor cells were seeded in 75 cm^2^ flasks at 950000, 2.8 millions, 1 million, 2.8 millions, 2.8 millions cells/flask for H1975, H1755, A549, H23, H1437, respectively. The different NSCLC lines used account for most of the mutational diversity and tumor origin found in NSCLC patients (A549: carcinoma mutated *KRAS*, *STK11*, *TP53*; H1755: stage 4 adenocarcinoma mutated *BRAF*, *TP53*; H1437: stage 1 adenocarcinoma mutated *TP53*; H23: adenocarcinoma mutated *KRAS*, *STK11*, *TP53*; H1975: adenocarcinoma mutated *EGFR* ^*T790M*^, *EGFR* ^*L858A*^, *PIK3CA*, *TP53*). 12 mL of supplemented RPMI was added in each T75 flask at the beginning of the experiment, and each NSCLC culture media was collected from a single flask after 72 h, and centrifuged at 300 g, 5 min to remove any cell debris. NSCLC cells were counted to ensure reproducibility between conditions and experiments. NSCLC CM were prepared by adding 50% tumor cell culture medium and 50% supplemented EGM2 medium. HUVECs were seeded in 0.1% gelatin‐coated 6‐well plate with 100% supplemented EGM2 medium, and treated with NSCLC CM on both the following day (day 0) and the day after (day 0 + 24 h). The treatments were then stopped after 48 h (day 0 + 48 h) or 72 h (day 0 + 72 h). A combination of TGF‐β2 (130–123‐657; Milteny) (10 ng/mL) and IL‐1β (130‐093‐897; Milteny) (10 ng/mL) was used as EndMT positive control. Seemingly to NSCLC CM, the TGF‐β2/IL‐1β combination was renewed at day 0 and day 0 + 24 h. Secretome from the normal bronchial epithelial cell line BEAS‐2B was used as a negative control. After 72 h of culture, the BEAS‐2B culture media was collected and centrifuged at 300 *g*, 5 min to remove any cell debris. It was further diluted in supplemented EGM2 medium to normalize to a similar number of producing cells when compared to NSCLC CM.

### 2.2 Viability Assay

HUVECs were cultured in 96‐well plates at 5,000 cells/well. After treatment, medium was replaced by 50 μL EGM‐2 completed with 50 μL CellTiter‐Glo (Promega). After shaking, the plate was incubated at room temperature for 10 min and luminescence was read with CLARIOstar equipment.

### 2.3 Immunofluorescence

HUVECs were cultured in 24‐well plates at 15,000 cells/well on gelatin‐coated glass coverslips. After treatment, the medium was removed, and cells were washed with 1× PBS and fixed with 4% paraformaldehyde (PFA) for 30 min. Cells were permeabilized with 0.5% Triton + 2% BSA for 2 h. Cells were first incubated with vWF antibody (ab6994; Abcam) and then with the secondary antibody anti‐rabbit Alexafluor 647 (A21245; Thermo Fisher Scientific). Moreover, cells were treated with phalloidin 488 (A12379; Invitrogen) and Hoechst 33342 (14,533; Sigma aldrich). Observations were realized with a confocal microscope using 60× magnification. Three pictures/well were analyzed using Fiji/ImageJ software.

### 2.4 Proliferation Assay

HUVECs were cultured in 96‐well plates at 5,000 cells/well. After treatment, medium was removed and 50 μL of Cyquant probe (C35006; Thermo Scientific) were added in each well. After an incubation for 45 min at 37°C and 5% CO_2_, the plate was read with a spectrophotometer (SpectroMax M2) at 485 nm excitation and 530 nm emission.

### 2.5 Sprouting Assay

HUVECs were cultured in 6‐well plates at 120,000 cells/well for the condition “48 h” and 100,000 cells/well for the condition “72 h”. After treatment, 40 μL of ECM gel (Corning) was plated and 15,000 cells/well were seeded. The sprouting was allowed to proceed for 6 h. Formation of sprouting networks was observed with a micropatterning microscope using 4× magnification. Three pictures/well were analyzed using ImageJ software with the angiogenesis analyzer plugin developed by Carpentier et al. [22].

### 2.6 Migration

HUVECs were cultured in 6‐well plates at 120,000 cells/well for the condition “48 h” and 100,000 cells/well for the condition “72 h”. After incubation, 4,000 cells/well were seeded with their respective treatments (TGF‐β2/IL‐1β combination and NSCLC CM) and placed in the IncuCyte at 37°C and 5% CO_2_. The migration was observed with IncuCyte microscope using 10× magnification. The migration was allowed for 24 h with 5 pictures/well every hour. Pictures were analyzed using Fiji/ImageJ software.

### 2.7 Flow Cytometry

HUVECs were cultured in 6‐well plates at 120,000 cells/well for the condition “48 h” and 100,000 cells/well for the condition “72 h”. After treatment, cells were fixed with 2% PFA + 1× PBS solution and then permeabilized with a 0.1% Tween20 + 1× PBS. Cells were incubated with AlexaFluor488 conjugated anti‐vWF (ab195028; Abcam) and APC conjugated anti‐α‐SMA (IC1420A; R&D Systems), or PE conjugated anti‐CD31 (FAB3567P; R&D Systems) and APC conjugated anti‐CD44 (338,806; Biolegend) antibodies for 30 min at room temperature. Analysis was conducted with BD FACS CantoTM II equipment (Biosciences), and measurements were processed with FlowJo V10 software.

### 2.8 Secretome Proteomic Analysis

#### 2.8.1 Digestions and LC–MS/MS Analyses Were Performed at the Prot'ICO Proteomics Facility

The cells were incubated for 72 h in RPMI with 10% SVFd and 1% PS. Supernatants were washed away with 1× PBS and replaced by RPMI without SVF and allowed to condition this new medium for 24 h. Cells were counted for subsequent normalization, and CM (~6 mL) were collected and centrifuged for 5 min at 300 *g* and concentrated on an amicon spin reverse 5 kDa/2 mL membrane filtration device. Proteins were denatured in 0.1% Rapigest SF acid‐labile detergent (Waters), 5 mM DTT, and 50 mM ammonium bicarbonate at 95°C for 30 min (200 μL final). Thiol residues were thus chemically reduced and subsequently cooled down to RT, then protected by alkylation in 20 mM MMTS (Sigma Aldrich) for 10 min at 37°C. 5 μg of trypsin (ABSciex) per sample were added and incubated at 37°C overnight. Peptides were then cleared by centrifugation, desalted on C_18_ sep‐pack reverse phase microcolumns as described in the “Stage‐tips” procedure, and peptides were eluted in Acetonitrile (ACN) [23]. Eluates were dried in a vacuum centrifuge concentrator (Thermo), resuspended in 25 μL of 10% ACN and 0.1% Formic Acid (FA). Eluate flow was electrosprayed into a timsTOF Pro 2 mass spectrometer (also from Bruker) for the 60 min duration of the hydrophobicity gradient ranging from 99% of solvent A (0.1% FA in milliQ‐grade H_2_O) to 40% of solvent B (80% ACN and 0.1% FA in mQ‐H_2_O). The mass spectrometer acquired data throughout the elution process and operated in data‐independent analysis mode (DIA) with PASEF‐enabled method using the TIMS‐Control v.3.1.4 software (Bruker). Samples were injected in batch replicate order to circumvent possible technical biases.

#### 2.8.2 LC–MS/MS Data Analysis

The raw data were extracted, normalized and analyzed using Spectronaut software v. 18.6.231227.55695 (Biognosys) in Direct‐DIA mode, which modelized elution behavior, mobility and MS/MS events based on the Uniprot/Swissprot sequence 2022 database of human proteins. Protein identification false discovery rate (FDR) was restricted to 1% maximum, with match between runs (MBR) option enabled and inter‐injection data normalization. The precursors' and fragments' mass tolerances were set to 15 ppm. Oxidation of methionines was set as variable modifications while thiol groups from cysteines were considered completely alkylated by the MMTS reagent (Methylation). A minimum of two ratios of peptides was required for relative quantification between groups. Protein quantification analysis was performed using Label‐Free Quantification (LFQ) intensities. The resulting protein LFQ values were compared between groups as per the principal component analysis (PCA). Namely the group consisting of H1975 (*n* = 5), H23 (*n* = 5) and H1437 (*n* = 5) samples were used as a comparison to the two other separated groups with A549 (batch 1 with *n* = 3 and batch 2 with *n* = 5) or H1755 samples (batch 1 with *n* = 3 and batch 2 with *n* = 5). Of note, we did not observe any significant variations between the two batches of samples (cf. PCA plot). Differentially upregulated proteins were visualized with the EnhancedVolcano R function. Venn diagram was used to identify congruent differentially upregulated proteins (LogFC > 1, adjusted *p* value < 0.05) between H1755 vs. H1975 + H23 + H1437 and A549 vs. H1975 + H23 + H1437. Pathway level analysis was performed using the fgseaMultilevel function with adjusted *p* value < 0.05, only considering pathways with more than 3 proteins. MSigDB Hallmark 2020 and GO_Biological_Process_2023 were interrogated. Correlation heatmap was generated with the pheatmap function on the scaled expression of proteins from the “MSigDB Hallmark 2020—Epithelial Mesenchymal Transition”, “Extracellular Matrix Organization—GO:0030198” and “Collagen Fibril Organization—GO:0030199”. The mass‐spectrometry proteomics data have been deposited to the ProteomeXchange Consortium via the PRIDE [24] partner repository with the dataset identifier PXD054295 (https://www.ebi.ac.uk/pride/archive/projects/PXD054295).

### 2.9 Patient Survival and Correlation Analysis

*SPP1* expression was generated using KM‐plotter [25], an online integrated database that combines multiple independent datasets that regroup lung cancer patient transcriptomic data from GEO and TCGA databases. The following parameters were used: median *SPP1* (218058_at) expression, median survival, and Cox univariate regression (*n* = 1161 patients). Correlation analysis was performed using GEPIA 2 [26] between *SPP1* and *PECAM1* in LUAD tumor, LUAD normal, and Lung GTEx datasets. The Spearman correlation index was calculated.

### 2.10 Statistical Analysis

Statistical analyses were performed with GraphPad Prism 8.3 software (GraphPad Software LLC). To compare the different conditions at a given time, the non‐parametric Kruskal–Wallis test was used, followed by Bonferroni correction (post hoc Dunn test) when appropriate. Comparisons between a condition at different times were assessed using the non‐parametric Mann–Whitney test.

## 3 Results

### 3.1 Cancer Cell Secretomes Have no Effect on Endothelial Cell Viability, Proliferation, and Tube Formation

To better understand how cancer cells can remodel the vascular compartment, we sought to discover the impact of cancer cell secretomes on EC biology. Since the cancer cell lines tested are grown in different media than ECs (RPMI versus EGM2), and since endothelial cells are sensitive to media composition [27], we first investigated the effect of a 50:50 mixture of RPMI:EGM2 on ECs. Compared to EGM2, treatments for 48 h and 72 h with RPMI:EGM2 did not alter cell viability (Figure S1), proliferation (Figure S1), their ability to form capillaries on ECM gel (Figures S1), or their morphology (Figure S1). Similarly, there was no difference in EC migration after treatment with either condition (Figures S1). Therefore, we selected EGM2 as the control condition for the remaining experiments.

**FIGURE 1:**
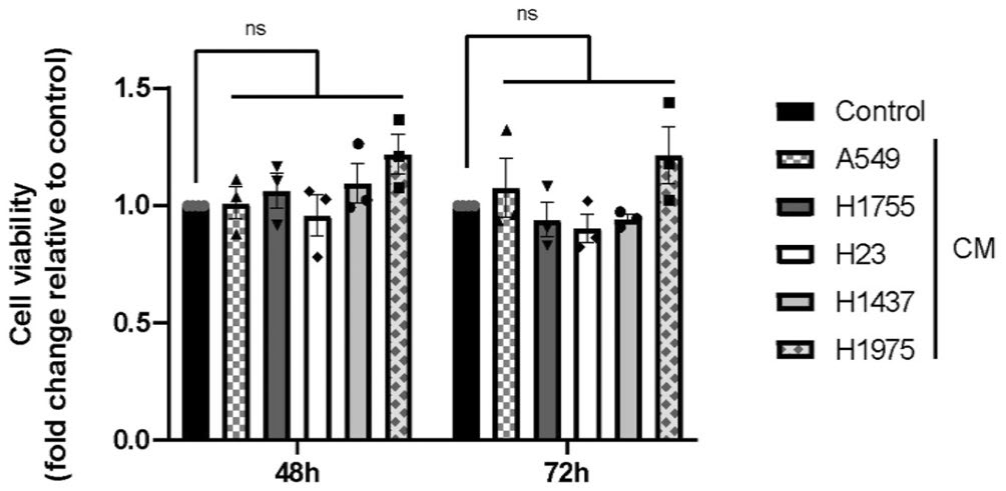
Assessing EC viability using different NSCLC secretomes. Graphical representation of CellTiter‐Glo luminescence‐based viability test. Results are reported as fold change to EGM2 100%. Graph bars show the mean ± SEM. *N* = 3 independent experiments. CM, conditioned media.

**FIGURE 2:**
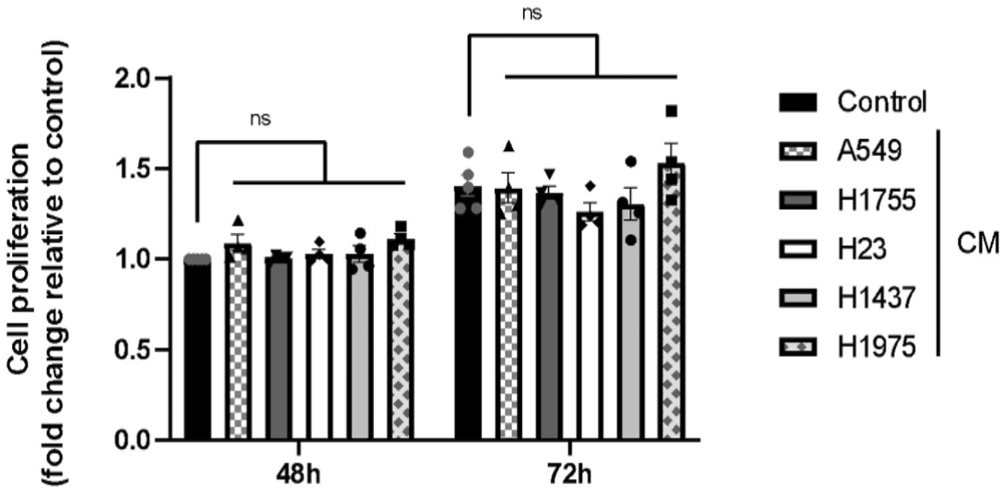
Evaluation of EC proliferation after treatment with different CMs. Graphical representation of Cyquant fluorescence‐based proliferation test. Results are reported as fold change to EGM2 100% after 48 h treatment. Graph bars show the mean ± SEM. *N* = 4 independent experiments.

**FIGURE 3:**
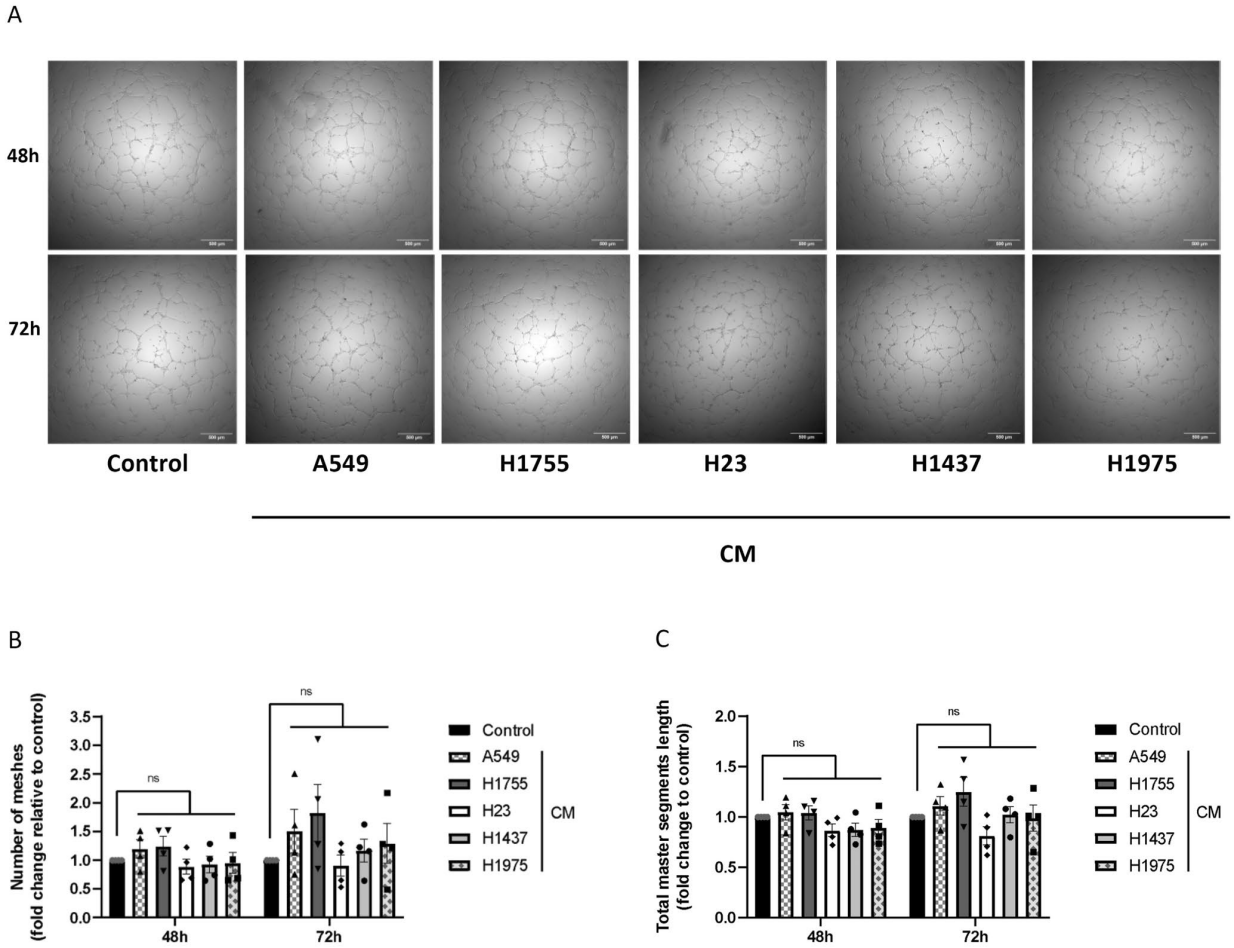
Analysis of capillary formation capacity of secretome‐treated HUVECs (A). Graphical representation of (B) the number of meshes and (C) the total master segments length. The results represent fold changes compared to the EGM2 100% condition. Graph bars show the mean ± SEM. *N* = 4 independent experiments.

No significant decrease in cell viability was observed after treatment with all CM after 48 and 72 h compared to the control condition (Figure 1). Seemingly, after 48 h of treatment, no change in EC proliferation was observed compared to the control condition. Furthermore, although EC proliferation increased significantly between 48 and 72 h (whatever the treatment conditions), after 72 h there was no difference in proliferation between each treatment condition compared with the control condition (Figure 2). Tubulogenesis was performed using ECs treated with each of the CMs prior to their seeding on a 3D matrix (Figure 3A). After 48 h of treatment, no difference in the number of meshes (Figure 3B) or in the total master segments length was observed (Figure 3C). However, after 72 h of treatment, a slight increase in the number of meshes (Figure 3B) and the total master segments length (Figure 3C) was found in cells treated with CM from A549 and H1755 compared to the control condition. This increase was not found in the other conditions.

### 3.2 Endothelial Cells Treated With Different Cancer Cell Secretomes Adopt a Partial Endothelial‐to‐Mesenchymal Phenotype

In response to various *stimuli*, ECs can undergo the EndMT that fosters tumor cell progression, aggressiveness, and treatment resistance [10]. Among these different *stimuli*, cytokines such as TGF‐β2 and IL‐1β effectively induce EndMT [28, 29] and have been chosen as positive controls in this study. While a significant decrease in viability and proliferation was observed in TGF‐β2/IL‐1β‐treated HUVECs compared to control cells after both 48 and 72 h of treatment, there was only a slight reduction in their ability to form stable tubular structures (Figure S2).

**FIGURE 4:**
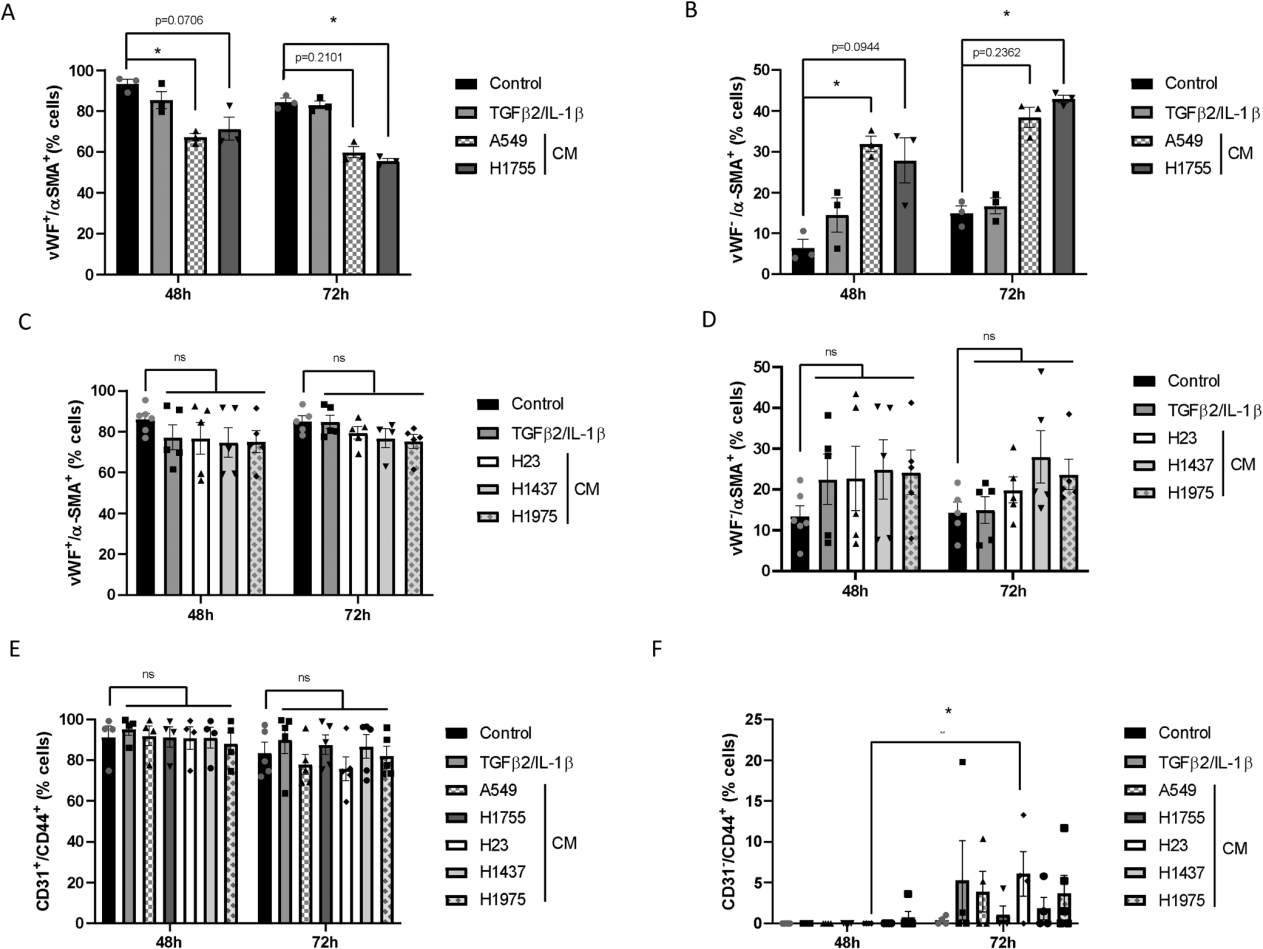
Evaluation of the expression of endothelial or mesenchymal markers in secretome‐treated HUVECs by flow cytometry. Analysis of the percentage of (A) vWF^+^/α‐SMA^+^ and (B) vWF^−^/α‐SMA^+^ cells from A549 and H1755 treated cells, (C) vWF^+^/α‐SMA^+^ and (D) vWF^−^/α‐SMA^+^ cells from H23, H1437 and H1975 treated cells, (E) CD31^+^/CD44^+^ cells from HUVECs treated with each secretome and (F) CD31^−^/CD44^+^ cells from HUVECs treated with each secretome. Graph bars show the mean of percentage ± SEM, *p < 0.05. *N* = 3–5 independent experiments.

In addition to the functional and physiological disruption of phenotypic characteristics, EndMT is also characterized by a subtle change in the expression of protein markers [20]. Again, we first ruled out any difference between EGM2 and the RPMI:EGM2 mixture in terms of endothelial (vWF) or mesenchymal (α‐SMA) marker expression by flow cytometry (Figure S3A,B). After both 48 and 72 h of treatment with TGF‐β2/IL‐1β, no change was observed in the percentage of vWF^+^/α‐SMA^+^ ECs as compared to the control condition. However, we observed a decrease in the percentage of vWF^+^/α‐SMA^+^ cells treated with CMs from A549 and H1755 after 48 and 72 h of treatment (Figure 4A). Interestingly, this decrease was not observed in HUVECs treated with CMs from H23, H1437, or H1975 (Figure 4C).

At 48 h, treatment with TGF‐β2/IL‐1β induced an expected increase in the percentage of vWF^−^/α‐SMA^+^ cells compared to the control condition, although not significant. Surprisingly, in comparison, HUVECs treated with CMs from A549 or H1755 induced a strong increase in the percentage of vWF^−^/α‐SMA^+^ cells after 48 and 72 h of treatment (Figure 4B; Figure S3C,D). This increase was statistically significant for HUVECs treated with CM from A549 at 48 h and from H1755 at 72 h compared to the control condition (Figure 4B). Although the percentage of vWF^−^/α‐SMA^+^ cells was induced, the results appear less pronounced in HUVECs treated with CM from H23, H1437, or H1975 (Figure 4D).

Recently, CD44 variants have been linked to the EndMT in the context of pulmonary hypertension [30]. We, therefore, investigated a second pair of markers, using CD31 and CD44 as endothelial and mesenchymal markers, respectively. We showed that none of the treatments modified the percentage of CD31^+^/CD44^+^ cells at 48 h, whereas a slight decrease in the level of CD31^+^/CD44^+^ cells was observed at 72 h in HUVECs treated with CM from A549 and H23 compared to the control condition (Figure 4E). Although the level of expression of CD31^−^/CD44^+^ cells was practically zero after 48 h, regardless of the treatment, we observed an increase in the percentage of CD31^−^/CD44^+^ cells in the presence of TGF‐β2/IL‐1β and the different CM compared to the control condition (Figure 4F). Although most cells treated with CM retain expression of the endothelial marker CD31, a small fraction begin to express the mesenchymal protein CD44.

### 3.3 Secretome Increases Stress Fiber Expression and Alters Endothelial Cell Morphology

**FIGURE 5:**
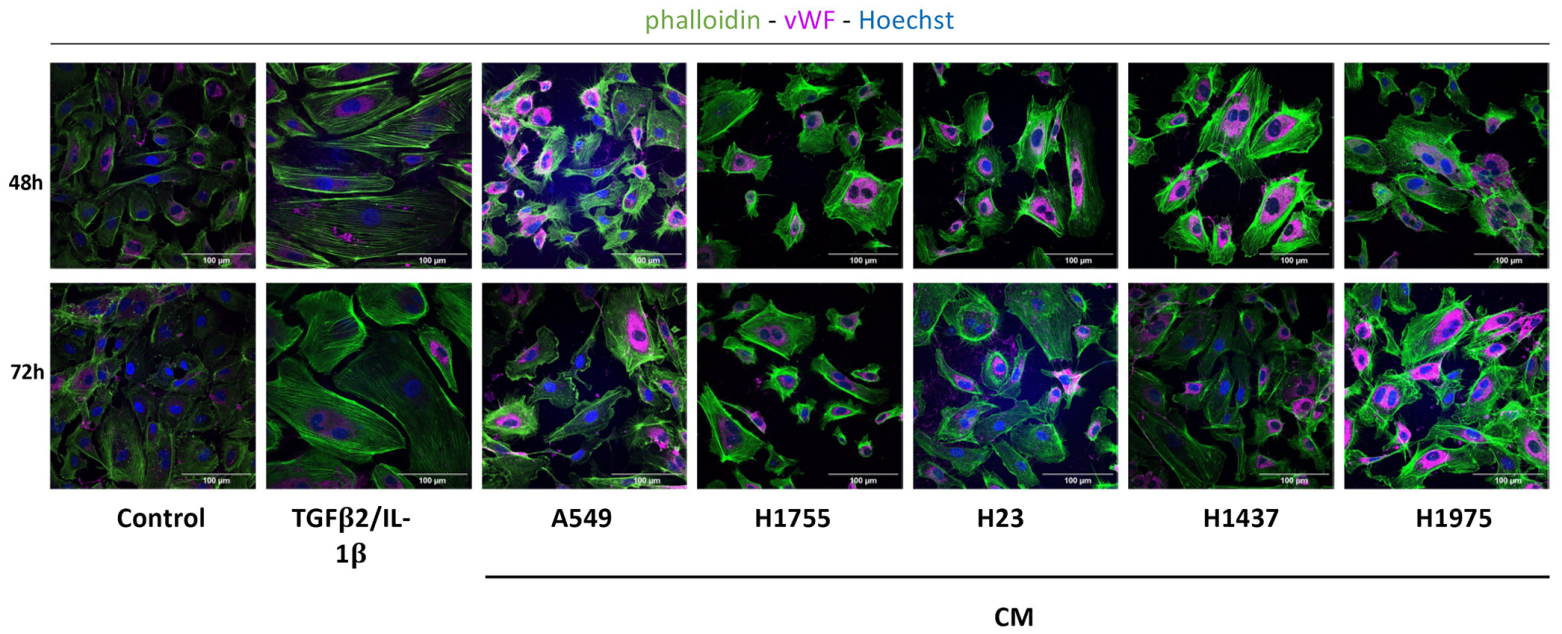
Analysis of stress fiber expression and EC morphology in HUVECs treated cells. Confocal microscopy analysis of HUVEC treated for 48 h or 72 h with TGFβ2/IL‐1β or different CM. Nucleus are counterstained with Hoechst (blue). Actin stress fibers (phalloidin, green) and the endothelial protein vWF (magenta). Scale bar = 100 μm. Representative images from *N* = 4 independent experiments.

The EndMT process is usually associated with cytoskeleton remodeling and a change in morphology, a prerequisite for the pro‐migratory phenotype of mesenchymal and EndMT‐transiting ECs [31]. Confocal microscopy observations showed an altered cell morphology with elongated and enlarged cells after 48 and 72 h of treatment with TGF‐β2/IL‐1β or each cancer cell CM compared to the control condition (Figure 5). In addition, phalloidin staining in TGF‐β2/IL‐1β‐treated HUVECs at both treatment times revealed a striking increase in the number of thick stress fiber bundles, which were generally aligned along the major cell axis. We observed a similar phenotype in all CM treatment conditions (Figure 5).

### 3.4 Secretomes Increase the Migration of Cells Whose Phenotype Is Altered

**FIGURE 6:**
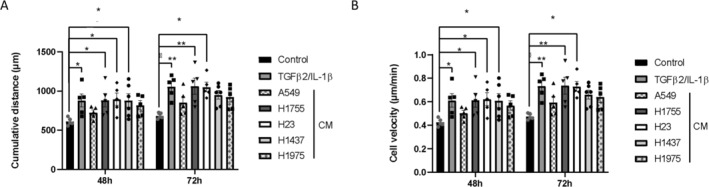
Analysis of spontaneous migration of CM‐treated HUVEC treated. Graphical representation of (A) cumulative distance and (B) velocity of HUVECs treated with each CM. Results are reported as fold change in comparison with control condition. Graph bars show the mean ± SEM, *p < 0.05; **p < 0.01. *N* = 5 independent experiments.

In close correlation with cell shape and morphology, we then sought to characterize the effect of cancer cell secretomes on EC migration. At 48 and 72 h, a significant increase in cumulative distance and cell velocity was found in both TGF‐β2/IL‐1β‐treated cells and H1755‐, H23‐, and H1437‐treated cells compared to control cells (Figure 6A,B). Although not statistically significant, a similar increase was observed at both treatment times in cells treated with CM from A549 and H1975 compared to control cells (Figure 6A,B).

### 3.5 Proteomic Analysis of the Secretomes Revealed Mediators of the EndMT

**FIGURE 7:**
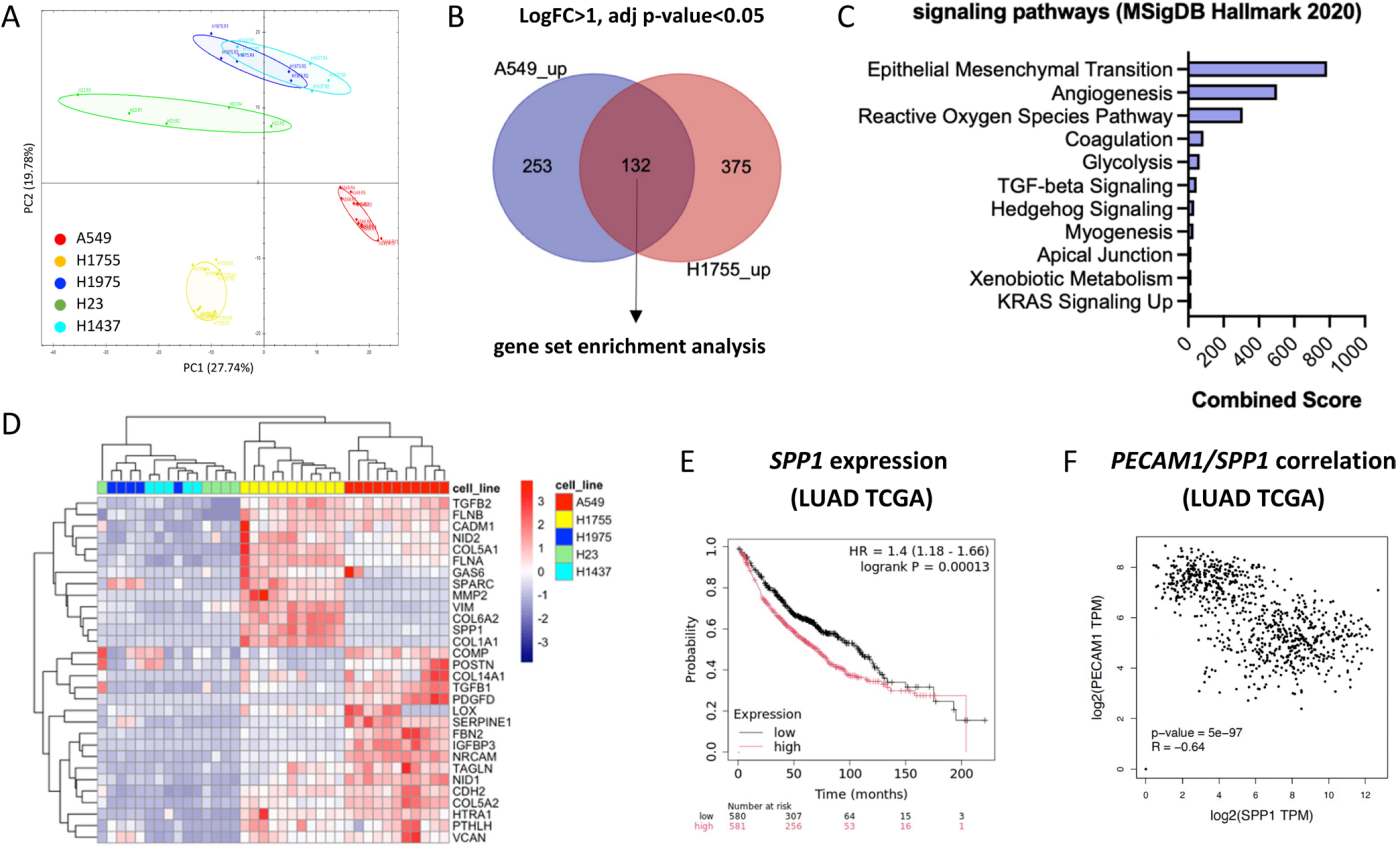
Mass‐spectrometry protein analysis of the secretome from NSCLC tumor cell lines. Schematic representation of (A) the principal component analysis (PCA) of the various samples analyzed (in red A549 *n* = 11, in yellow H1755 *n* = 11, in blue H1975 *n* = 5, in light blue H1437 *n* = 5, in green H23 *n* = 5). (B) Differential expression analysis between A549 or H1755 vs. H1437 + H1975 + H23, and the differentially upregulated proteins (LogFC > 1, adjusted *p* value < 0.05) are plotted in a Venn diagram. (C) Gene set enrichment analysis is performed on the 132 proteins, congruently upregulated in A549 and H1755. All depicted pathways have an adjusted *p* value < 0.05. (D) Correlation heatmap of key proteins involved in EMT and matrix remodeling, and upregulated in A549 and H1755 secretome. (E) Kaplan–Meier representation of TCGA lung adenocarcinoma (LUAD) patient survival according to the *SPP1* expression. (F) Correlation analysis between *PECAM1* (CD31) and *SPP1* expressions in LUAD TCGA. R shows the Spearman correlation index.

Secretomes from the different NSCLC lines tested mediate various phenotypic alterations of exposed ECs, with A549 and H1755 inducing the most pronounced effects. Importantly, compared to TGF‐β2/IL‐1β combination treatment, the CM from a healthy human bronchial epithelial cell line did not significantly alter their morphology, nor key endothelial and mesenchymal markers expressed by HUVECs after 72 h (Figure S4). Hence, suggesting that malignant alterations acquired by NSCLC lines translate in an altered secretome that could mediate the EndMT. We thus screened by mass‐spectrometry analysis the composition of the NSCLC CMs, and principal component analysis (PCA) identified three main groups of samples: a first group encompassing mixed samples from H1975, H23, and H1437 cells; a second with H1755 samples; and a third with A549 samples (Figure 7A). On this basis, differential analysis was performed to highlight deregulated proteins in H1755 or A549 compared to the H1975 + H23 + H1437 group (Figure S5A). Among the significantly upregulated proteins from H1755 and A549 (Log FC > 1, adjusted *p* value < 0.05), 132 proteins were found in common (Figure 7B) and relate to the epithelial‐to‐mesenchymal transition (or EndMT in ECs), angiogenesis, and reactive oxygen species signaling pathways, which rank as the three most prevalent pathways (Figure 7C, Figure S5B). Gene ontology analysis also pinpointed key biological processes involving extracellular matrix and collagen fibril organization (Figure S5C), further shedding light on the matrix‐remodeling potential of A549 and H1755 secretomes. Correlation heatmap representation of the top deregulated proteins involved in the EndMT (e.g., TGFß1/2, SPARC, SPP1, MMP2, SERPINE1, TAGLN, VIM) and matrix‐remodeling signaling pathways (e.g., COL1A1/5A1/5A2/6A2/14A1, NID1/2, POSTN, LOX) further confirmed the sample group distribution identified by PCA (Figure 7D). Notably, different protein expression patterns were distinguished between the A549 and H1755 secretomes. Finally, we selected the most commonly upregulated target in A549 (3.02 Log2FC) and H1755 (5.74 Log2FC), namely the SPP1/Osteopontin, whose expression was correlated to poor survival in patients with lung adenocarcinoma (LUAD) (Figure 7E). Interestingly enough, *SPP1* expression was inversely correlated to *PECAM1* (encoding for the endothelial marker CD31) in tumor and healthy LUAD TCGA samples (Figure 7F).

## 4 Discussion

The aim of the present study was to investigate the effect of conditioned media from NSCLC tumor cell lines on the EC phenotype. Interestingly, a modification of the endothelial phenotype was found when HUVECs were treated with different CMs from different NSCLC cell lines. This phenotypic change was associated with a morphological change, an increase in both stress fiber expression and spontaneous migration. Furthermore, an increase in mesenchymal markers (α‐SMA and CD44) confirmed the phenotypic changes. However, the secretomes did not significantly reduce the expression of endothelial markers (vWF) and did not modify the rate of double‐labeled cells (vWF^+^/α‐SMA^+^ or CD31^+^/CD44^+^) because it remained high. Taken together, these data suggest that CMs are capable of inducing partial EndMT.

ECs are known for their plasticity during angiogenesis [32, 33] or EndMT [34, 35]. EndMT was first described in cardiac fibrosis and pulmonary hypertension [36]. Its involvement in NSCLC was demonstrated in a study investigating the effect of conditioned media on HUVECs, with an increase in resistance to anticancer treatments in lung cancer cell lines. Dynamic interactions involving both cancer cells and vascular ECs have been proposed to explain the induction of EndMT. Using 2D and 3D models, the authors confirmed crosstalk between tumor cells and ECs to induce phenotypic changes [37]. In addition, another study in uterine squamous cell carcinoma showed that PAI‐1/SERPINE1 CAFs promoted the differentiation of lymphatic ECs into mesenchymal cells, again highlighting the close interactions between tumor and ECs to induce EndMT [38]. The data from these studies justify the experimental models chosen for our study. Under these conditions, we showed a morphological change associated with an increase in both stress fiber expression and cell migration. Taken together, these data suggest a phenotypic transition of CM‐treated EC in favor of a mesenchymal phenotype as it has been described by several authors. For instance, Pinto et al. showed that the acquisition of an invasive phenotype in TGF‐β2‐induced EndMT is mediated by a SNAIL signaling pathway [39]. Recently, this has been extensively characterized at the single cell level by combining different EndMT models on HUVECs (PMID: 39121218) [40]. Similarly, it has been demonstrated by Ma et al. an increase in the migratory capacity of mouse pulmonary vascular endothelial involved in EndMT induced by PM_2.5_ particles [41].

To confirm this NSCLC secretome‐induced differentiation of ECs, the expression of various mesenchymal and endothelial markers was assessed by flow cytometry. EndMT is characterized by a loss of expression of endothelial markers in favor of mesenchymal markers. In this study, two pairs of endothelial and mesenchymal markers were tested to avoid the variability caused by the type of vascular cells used [42]. Interestingly, the secretome of two NSCLC cell lines appeared to alter the expression of the markers tested. The secretome of A549 and H1755 cells induced an increase in the expression of vWF^−^/α‐SMA^+^ cells and a decrease in the expression of vWF^+^/α‐SMA^+^ cells. The same trends were confirmed, albeit to a lesser extent, in the study of the expression of the pair CD31/CD44. The absence of a clear reduction in double‐labeled cells in favor of cells significantly expressing mesenchymal markers suggests that, under the experimental conditions chosen, NSCLC secretomes induce partial EndMT. It is necessary to consider the chosen experimental protocol to explain this partial EndMT. In fact, while studies have confirmed robust EndMT [43] characterized by phenotypic changes in ECs after at least 1 week of treatment [28], we chose to treat HUVECs with tumor cell secretomes for a maximum of 72 h. This choice was based on preliminary studies conducted by our team [44] in which it has been confirmed the absence of toxicity of tumor cell secretomes towards endothelial cells. Furthermore, in this study the analysis of EndMT was conducted on ECs cultured in 2D while the use of 3D spheroid models seems to be more favorable for the induction of EndMT. In recent years, a paradigm shift from 2D to 3D cell culture techniques has occurred, because 2D cell culture involves growing cells in a flat dish, which can lead to the formation of unnatural cell attachments. Further, simplifying the 2D assay system does not provide us the data that would be utilized in translational research. Hence, Kim et al. conducted comparative studies of the effects of secretomes on the activation of EndMT in HUVECs in 2D and 3D cultures of NSCLC cells and confirmed that the increase in certain growth factors in NSCLC‐3D culture conditions may lead to the activation of the EndMT process in HUVECs [37]. Therefore, we acknowledge the limitations of our study and it will be appropriate to plan further experiments to assess the EndMT capacity of ECs derived from different vascular beds and incorporate them into multicellular 3D tumor spheroid models.

Other interesting hypotheses regarding partial EndMT have been proposed in a recent study by Takahashi et al. [13]. Thus, they suggested that EndoMT is closely associated with tumor progression through the formation of CAFs and angiogenesis. CAFs originated from the cells that have undergone full EndoMT, and they proposed that those cells cannot reverse to ECs, while the partial EndoMT state is a reversible phenotype allowing cells to regain their endothelial characteristics [35]. This retrieval of endothelial characteristics was confirmed in our study, following the increase in the capacities of ECs treated with A549 and H1755 cell secretomes to form capillaries on Matrigel after 72 h of incubation.

In order to identify NSCLC‐secreted mediators of the EndMT, our mass‐spectrometry analysis provides an interesting dataset of targets that could potentially induce or modulate this process. Some proteins appear highly upregulated in specific cell lines; for instance, this is the case for the pro‐EndMT PAI1/SERPINE1, which is enriched by a 2.7‐fold change in A549 compared to H1975/H23/H1437. As these NSCLC present specific genomic alterations (representative of NSCLC patient's heterogeneity), it will be highly relevant to identify tumor signatures that could be correlated to the EndMT levels in tumors and patient response to therapies. We showcased the example of SPP1, which has been linked to EMT in various cancers, including NSCLC [45], but to our knowledge, a limited number of studies exist on SPP1 and EndMT in cancer [46, 47] and should thus be further studied. Interestingly, the PECAM1/SPP1 correlation analysis indicates that healthy lung tissues are mostly PECAM1^high^SPP1,^low^ while tumor samples appear PECAM1^low^SPP1^high^ (data not shown). One could hypothesize that it may highlight a high occurrence of EndMT in NSCLC adenocarcinoma tumors, but this has to be validated.

## Author Contributions

**Clara Bourreau:** investigation (lead), methodology (lead), writing – original draft (lead). **Emilie Navarro:** investigation (equal), methodology (equal), visualization (equal). **Marine Cotinat:** methodology (equal), visualization (equal). **Morgane Krejbich:** methodology (equal), visualization (equal). **François Guillonneau:** investigation (equal), methodology (equal), writing – original draft (equal). **Catherine Guette:** investigation (equal), methodology (equal), writing – original draft (equal). **Alice Boissard:** methodology (equal). **Cécile Henry:** methodology (equal). **Isabelle Corre:** investigation (equal), methodology (equal), visualization (equal). **Lucas Treps:** conceptualization (lead), data curation (lead), formal analysis (lead), funding acquisition (lead), investigation (lead), methodology (lead), project administration (lead), resources (lead), software (lead), supervision (lead), validation (lead), visualization (lead), writing – original draft (equal), writing – review and editing (lead). **Nicolas Clere:** conceptualization (lead), data curation (lead), formal analysis (lead), funding acquisition (lead), investigation (lead), methodology (lead), project administration (lead), resources (lead), supervision (lead), validation (lead), visualization (lead), writing – original draft (equal), writing – review and editing (lead).

## Conflicts of Interest

The authors declare no conflicts of interest.

## Supporting information


**Figure S1.** Comparison of the control condition (EGM2) and the 50:50 mix condition on HUVEC. Graphical representation of (A) cell viability (*N* = 3 independent experiments), (B) cell proliferation (*N* = 4 independent experiments), (C) the analysis of the ability of EC to form capillaries on ECM gel with (D) the number of meshes and (E) the total master segments length on ECM gel (*N* = 4 independent experiments). (F) Confocal analysis of stress fiber (phalloidin) expression and EC morphology. Scale bar: 100 μm. Analysis of migration (*N* = 5 independent experiments) with (G) the cumulative distance and (H) the velocity of CM‐treated HUVECs. Graph bars show the mean ± SEM.
**Figure S2.** Comparison of the control condition and the TGF‐β2/IL‐1β treatment on HUVEC. Graphical representation of (A) cell viability (*N* = 3 independent experiments), (B) cell proliferation (*N* = 4 independent experiments) after TGF‐β2/IL‐1β treatment. Analysis of the ability of EC to form capillaries on ECM gel (C) with (D) the number of meshes and (E) the total master segments length (*N* = 4 independent experiments). Graph bars show the mean ± SEM, *p < 0.05; **p < 0.01.
**Figure S3.** Comparison of the control condition (EGM2) and the 50:50 mix condition on HUVEC marker expression. Analysis of the percentage of (A) vWF^+^/α‐SMA^+^, (B) vWF^−^/α‐SMA^+^, (C) CD31^+^/CD44^+^, (D) CD31^−^/CD44^+^ cells by flow cytometry (*N* = 3–5 independent experiments).
**Figure S4.** Effect of the conditioned media (CM) from a normal human bronchial epithelial cell line (BEAS‐2B) on HUVEC compared to the TGF‐β2/IL‐1β treatment. (A) Overall cell morphology was captured after 72 h of treatment. (B) Endothelial (*PECAM1*, *VWF*) and mesenchymal (*TAGLN*, *SNAI1*, *CDH2*) gene expression levels were studied by RTqPCR and normalized to the control condition (EGM2). (*N* = 3 independent experiments). Graph bars show the mean ± SEM, *p < 0.05; **p < 0.01.
**Figure S5.** Proteomic analysis of the NSCLC secretomes. (A) Differential analysis (DA) and (B) gene set enrichment analysis (GSEA) of H1755 or A549 vs H1975 + H23 + H1437 tumor cell secretomes. (C) GSEA of the congruent upregulated proteins between H1755 and A549 secretomes. All depicted pathways have an adjusted *p* value < 0.05.

## Data Availability

The proteomic data that support the findings of this study are openly available at https://fra01.safelinks.protection.outlook.com/?url=https%3A%2F%2Fwww.ebi.ac.uk%2Fpride%2Flogin&data=05%7C02%7CFrancois.Guillonneau%40ico.unicancer.fr%7C5cb64067c5d84f03807108dcae5e74fc%7C0d93e604f5f54880a9066eb91cc52ac4%7C0%7C0%7C638576967922858968%7CUnknown%7CTWFpbGZsb3d8eyJWIjoiMC4wLjAwMDAiLCJQIjoiV2luMzIiLCJBTiI6Ik1haWwiLCJXVCI6Mn0%3D%7C0%7C%7C%7C&sdata=rLptnQqr64yFdVpo02qBw9sa2XtVMUV8deSq1DRemkI%3D&reserved=0 with Username: reviewer_pxd054295@ebi.ac.uk and Password: WESqvK1SbciQ.
