# Supplementary material for: Secretomes From Non‐Small Cell Lung Cancer Cells Induce Endothelial Plasticity Through a Partial Endothelial‐to‐Mesenchymal Transition
Source: Cancer Med. 2025 Mar 3;14(5):e70707. doi: 10.1002/cam4.70707 (PMC11873768; doi:10.1002/cam4.70707)

Supplemental Figure 1

A

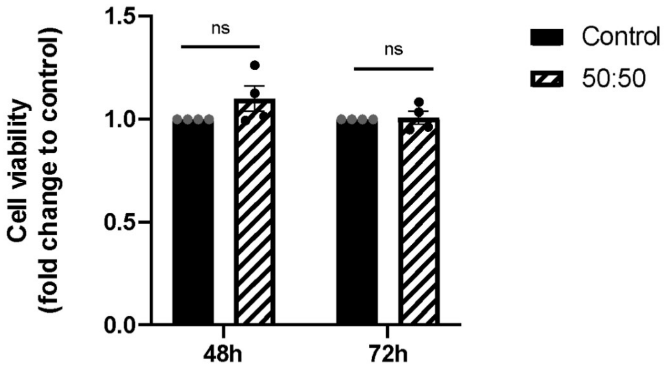

B

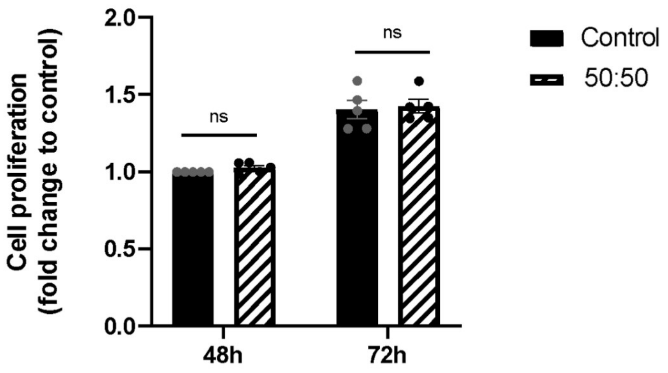

Supplemental Figure 1

C

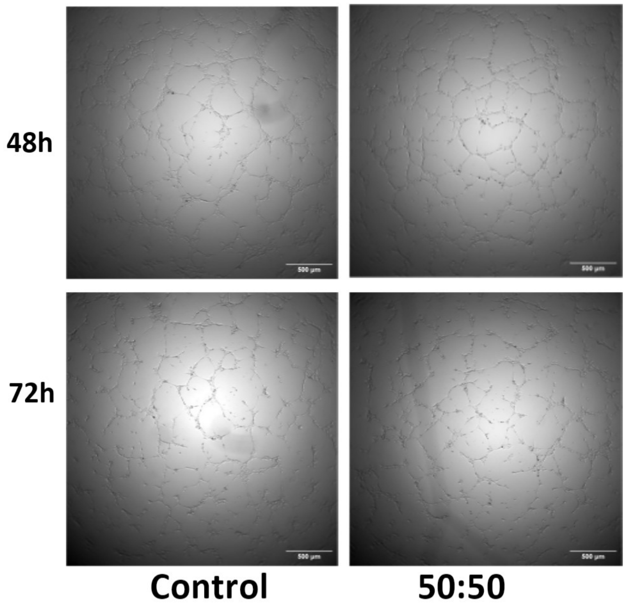

D

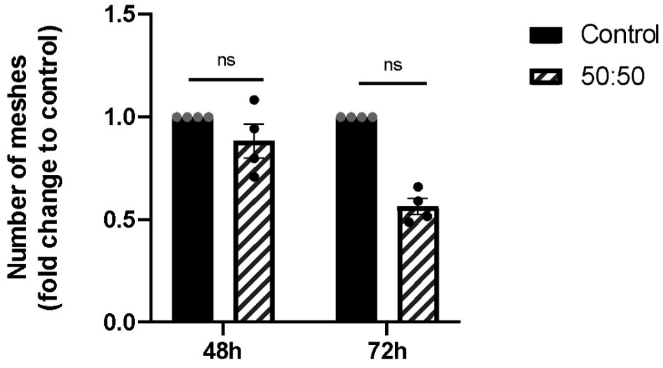

E

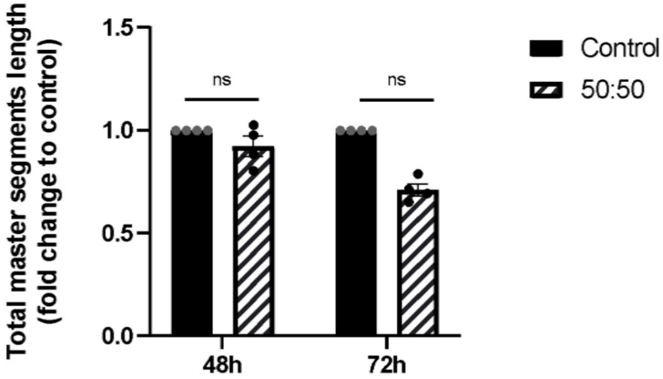

F

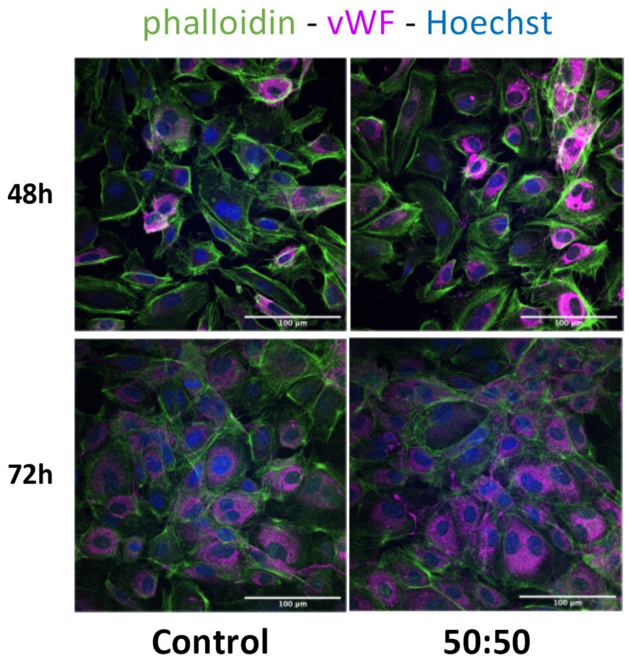
phalloidin - vWF - Hoechst
48h
72h
Control
50:50

G

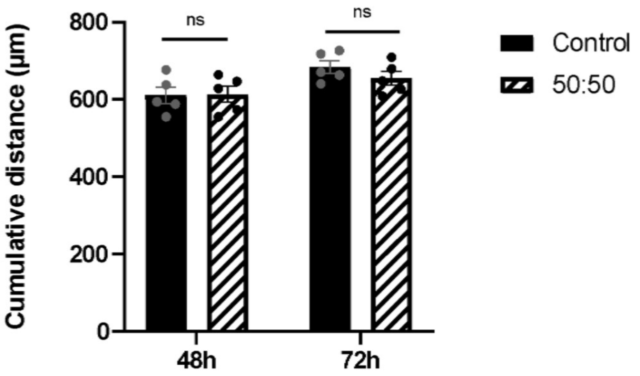
Cumulative distance (µm)
800
600
400
200
0
ns
ns
48h
72h
Control
50:50

H

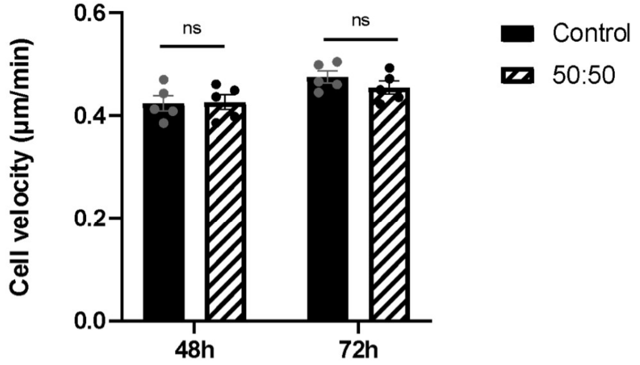
Cell velocity (µm/min)
0.6
0.4
0.2
0.0
ns
ns
48h
72h
Control
50:50

Supplemental Figure 2

A

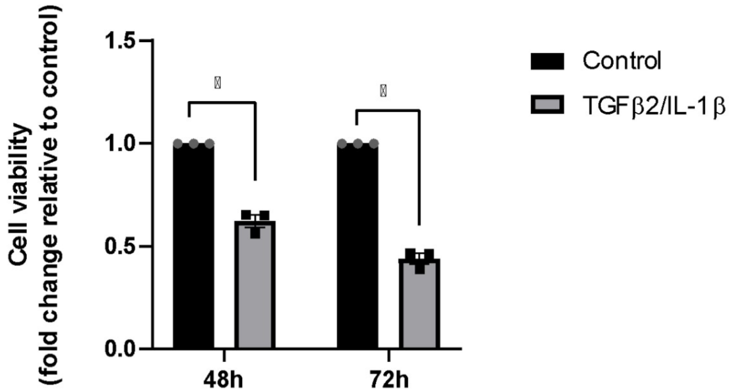

B

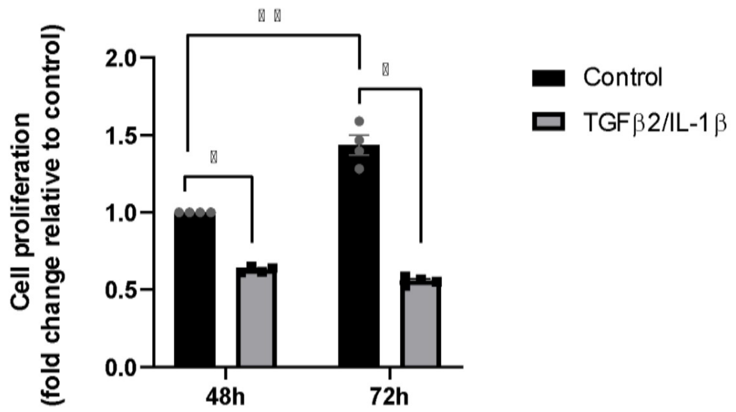

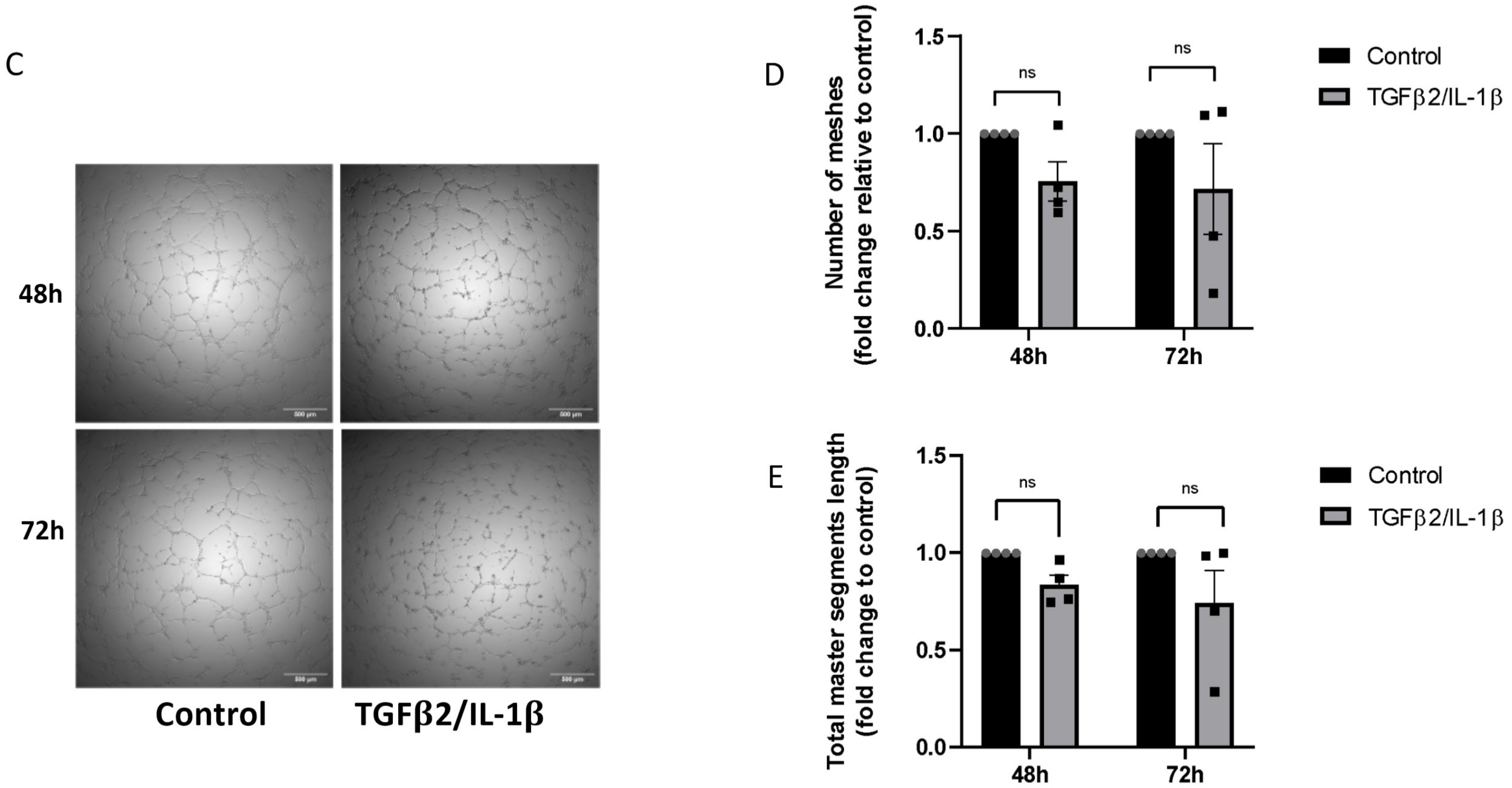
C
48h
72h
Control
TGFβ2/IL-1β
D
Number of meshes
(fold change relative to control)
1.5
1.0
0.5
0.0
ns
ns
48h
72h
Control
TGFβ2/IL-1β
E
Total master segments length
(fold change to control)
1.5
1.0
0.5
0.0
ns
ns
48h
72h
Control
TGFβ2/IL-1β

Supplemental Figure 3

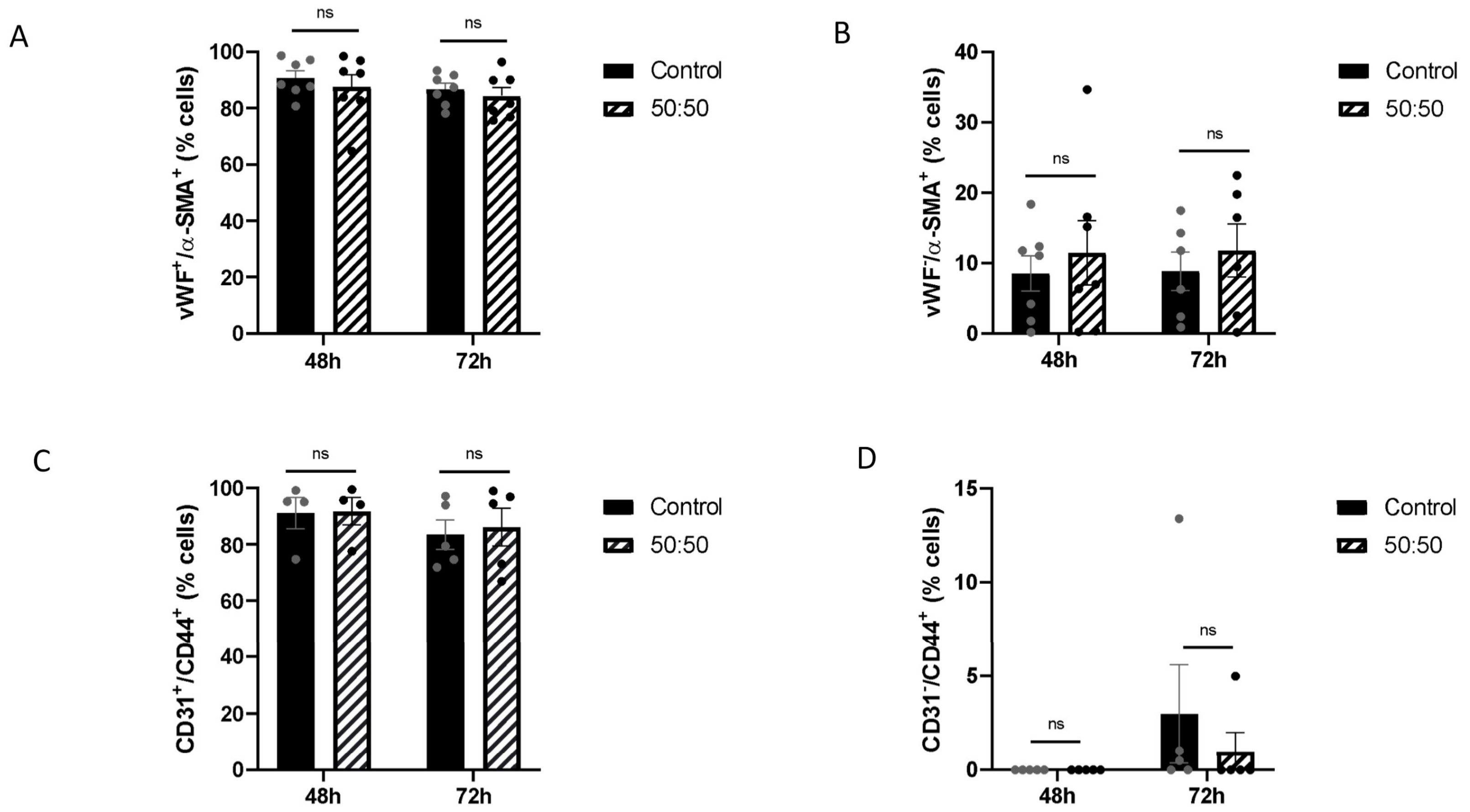

Supplemental Figure 4

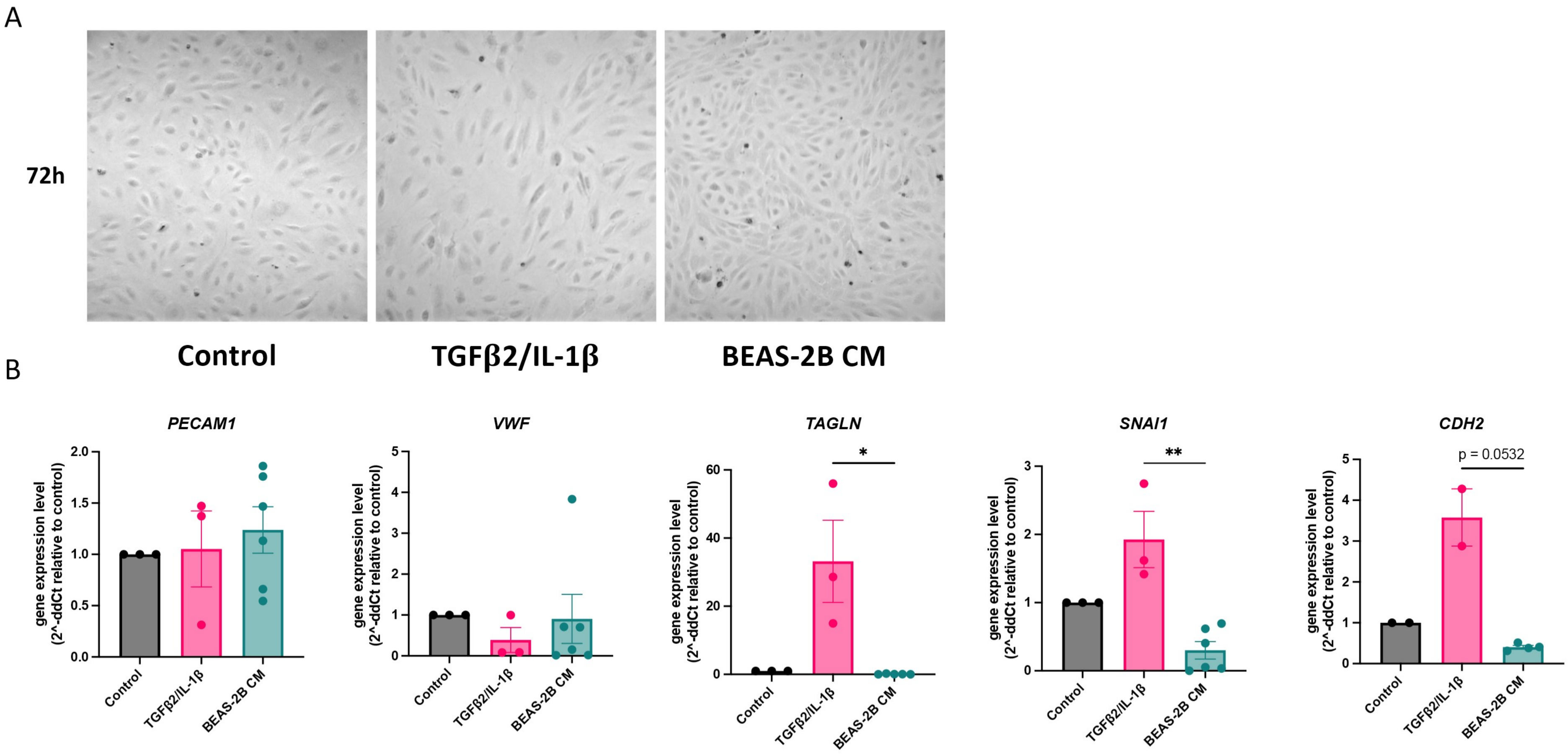

Supplemental Figure 5

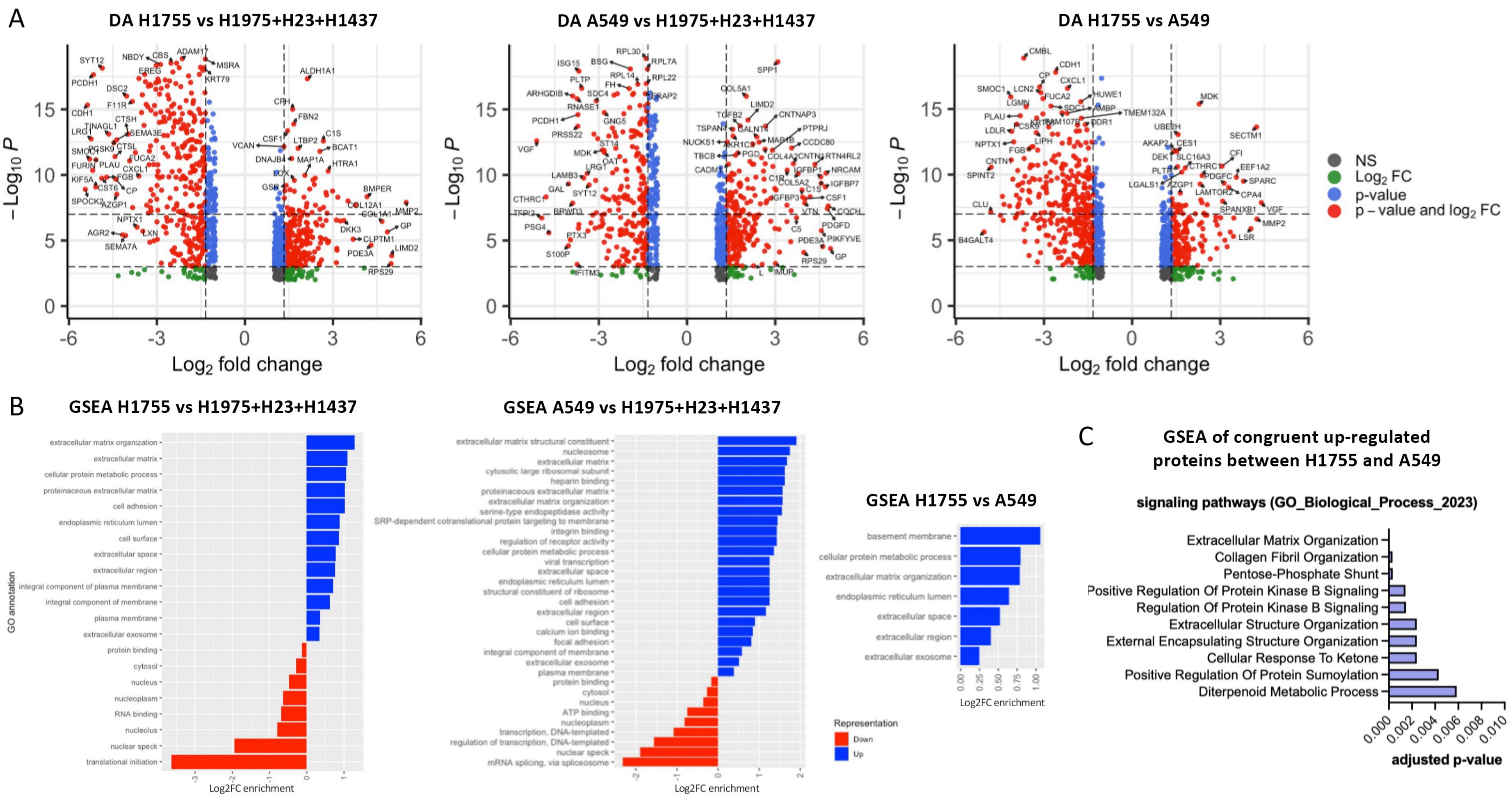

Supplement: Supplementary file 1 — Figure S1. Comparison of the control condition (EGM2) and the 50:50 mix condition on HUVEC. Graphical representation of (A) cell viability (N = 3 independent experiments), (B) cell proliferation (N = 4 independent experiments), (C) the analysis of the ability of EC to form capillaries on ECM gel with (D) the number of meshes and (E) the total master segments length on ECM gel (N = 4 independent experiments). (F) Confocal analysis of stress fiber (phalloidin) expression and EC morphology. Scale bar: 100 μm. Analysis of migration (N = 5 independent experiments) with (G) the cumulative distance and (H) the velocity of CM‐treated HUVECs. Graph bars show the mean ± SEM. Figure S2. Comparison of the control condition and the TGF‐β2/IL‐1β treatment on HUVEC. Graphical representation of (A) cell viability (N = 3 independent experiments), (B) cell proliferation (N = 4 independent experiments) after TGF‐β2/IL‐1β treatment. Analysis of the ability of EC to form capillaries on ECM gel (C) with (D) the number of meshes and (E) the total master segments length (N = 4 independent experiments). Graph bars show the mean ± SEM, *p < 0.05; **p < 0.01. Figure S3. Comparison of the control condition (EGM2) and the 50:50 mix condition on HUVEC marker expression. Analysis of the percentage of (A) vWF+/α‐SMA+, (B) vWF−/α‐SMA+, (C) CD31+/CD44+, (D) CD31−/CD44+ cells by flow cytometry (N = 3–5 independent experiments). Figure S4. Effect of the conditioned media (CM) from a normal human bronchial epithelial cell line (BEAS‐2B) on HUVEC compared to the TGF‐β2/IL‐1β treatment. (A) Overall cell morphology was captured after 72 h of treatment. (B) Endothelial (PECAM1, VWF) and mesenchymal (TAGLN, SNAI1, CDH2) gene expression levels were studied by RTqPCR and normalized to the control condition (EGM2). (N = 3 independent experiments). Graph bars show the mean ± SEM, *p < 0.05; **p < 0.01. Figure S5. Proteomic analysis of the NSCLC secretomes. (A) Differential analysis (DA) and (B) gene set enrichm [file CAM4-14-e70707-s001.pdf]
